# Insect-resistant rice and *Metarhizium anisopliae* sustain the brown planthopper susceptibility through AMPK-mediated lipid redistribution

**DOI:** 10.1007/s44297-026-00090-7

**Published:** 2026-11-14

**Authors:** Kai Lin, Yutao Hu, Guy Smagghe, Wenqing Zhang

**Affiliations:** 1 State Key Laboratory of Biocontrol and School of Life Sciences, Sun Yat-Sen University, Guangzhou, China; 2 Guangxi Key Laboratory of Agric-Environment and Agric-Products Safety, College of Agriculture, Guangxi University, Nanning, China; 3 Institute of Entomology, Guizhou University, Guiyang, China; 4 Department of Biology, Vrije Universiteit Brussel (VUB), Brussels, 1050 Belgium

**Keywords:** Brown planthopper, Lipid metabolism, Integrated pest management, Biological control, Insect adaptation, Energy trade-off

## Abstract

The brown planthopper (BPH), *Nilaparvata lugens*, rapidly overcomes host resistance and develops insecticide resistance, undermining sustainable rice pest control. Here, we show that combining insect-resistant rice IR36 with the entomopathogenic fungus *Metarhizium anisopliae* CQMa421 provides durable suppression of BPH under laboratory conditions. Across multiple generations, chemical insecticides drove resistance and host adaptation, whereas *M. anisopliae* maintained susceptibility. Combined exposure redistributed lipids by reducing diacylglycerol (DAG) and adenosine triphosphate (ATP) in the fat body and ovary while increasing DAG in the hemolymph, thereby prioritizing humoral immunity. This redistribution was mediated by AMPK activation in the fat body, which inhibited insulin signaling and lipid catabolism while limiting detoxification and reproduction. AMPK-mediated energy reallocation sustained BPH sensitivity to both resistant rice and fungal infection. Integrating plant resistance with microbial biocontrol therefore suppresses host adaptation and delays resistance evolution, offering a sustainable pest management strategy.

**Supplementary Information:**

The online version contains supplementary material available at https://doi.org/10.1007/s44297-026-00090-7.

## Introduction

The rapid growth of the global population has substantially increased food demand, making the reduction of pest-induced crop losses a key challenge for global food security. Agricultural pests remain one of the major constraints to crop productivity, particularly in intensive production systems where pest populations can rapidly adapt to control measures. Currently, three primary approaches dominate pest management strategies: pest-resistant crops, chemical insecticides, and microbial biopesticides [1]. Each approach provides important benefits but also presents limitations that challenge long-term sustainability.

Pest-resistant crops, developed through traditional breeding or genetic engineering, enhance plant defenses against specific pests and can significantly reduce pest damage while lowering reliance on chemical inputs. However, the durability of plant resistance is often threatened by the rapid adaptive evolution of herbivorous insects. Over-reliance on single resistance genes can accelerate the emergence of resistant pest populations, ultimately eroding control efficacy [2]. Chemical insecticides remain widely used because they provide rapid and reliable suppression of pest outbreaks. Nevertheless, their long-term effectiveness is compromised by the evolution of insecticide resistance, as well as by adverse impacts on non-target organisms, soil microbial communities, and ecosystem health. Furthermore, pesticide residues may pose risks to human health and food safety [3]. Microbial biopesticides, by contrast, exploit naturally occurring microorganisms to suppress pest populations and are generally characterized by high specificity, lower environmental impact and safety to natural enemies, making them increasingly attractive for sustainable pest management [4].

Over the past decades, integrated pest management (IPM) has been widely promoted as a strategy to reduce pesticide dependence while maintaining effective pest control. IPM emphasizes the integration of multiple complementary control tactics, including host plant resistance, biological control, and selective pesticide use, thereby minimizing environmental and health risks while maintaining agricultural productivity [5, 6]. Within IPM programs, insect-resistant crops have become a cornerstone strategy. However, in the ongoing evolutionary arms race between plants and herbivorous insects, pests frequently evolve physiological and behavioral mechanisms that allow them to overcome plant defenses [7].

A central mechanism underlying insect adaptation to plant defenses and insecticides involves metabolic detoxification. Herbivorous insects possess complex detoxification systems that enable them to metabolize toxic plant secondary metabolites and synthetic insecticides. These systems include cytochrome P450 monooxygenases (P450s), carboxylesterases (CarE), glutathione S-transferases (GSTs), UDP-glucuronosyltransferases (UGTs), and ATP-binding cassette (ABC) transporters, which together form a multi-layered biochemical defense network [8–11]. Elevated expression, amplification, or functional diversification of detoxification genes, such as members of the CYP450 family (including CYP9A), GSTs, and UGTs, has been shown to enhance the metabolic processing of insects toward plant defensive compounds and a wide range of insecticides, including nicotinoids, neonicotinoids, pyrethroids, and carbamates. These detoxification pathways play a crucial role in both insecticide resistance and host plant adaptation across multiple pest species [10, 12–19]. Consequently, understanding how pest detoxification systems respond to combined pest management strategies is essential for developing more durable control approaches.

Recent advances in biotechnology have renewed interest in the use of living microbial biopesticides as key components of IPM. Entomopathogenic fungi (EPF) and other microbial agents provide environmentally friendly pest suppression and may complement host plant resistance mechanisms [20]. However, despite their growing use, relatively little research has investigated the ecological and evolutionary outcomes of combining insect-resistant crops with living microbial biopesticides. Because these two approaches operate through fundamentally different modes of action, namely, plant-mediated chemical defenses versus pathogen-mediated infection, they may produce integrated effects that enhance pest suppression while potentially reducing the rate at which pests adapt to either strategy [21–23]. Indeed, recent evidence suggests that microbial biopesticides may reduce the likelihood that pests develop resistance to chemical insecticides and may also constrain adaptive evolutionary responses in pest populations [24]. Such effects are particularly relevant because the physiological mechanisms underlying adaptation to host plants often overlap with those responsible for insecticide resistance.

Among the physiological pathways that could mediate such trade-offs, energy metabolism has emerged as a central pathway. AMP-activated protein kinase (AMPK), a conserved heterotrimer of catalytic (α1/α2) and regulatory (β1/β2, γ1/γ2/γ3) subunits, serves as a key cellular energy sensor and regulator of metabolic homeostasis [25, 26]. AMPK is activated via two principal upstream routes—Ca^2^⁺-dependent CaMKKβ and AMP-dependent LKB1—both converging on Thr172 phosphorylation of the catalytic subunit; Tak1 can also phosphorylate this residue. AMP binding to the γ subunit further protects Thr172 from dephosphorylation, sustaining kinase activity. Through AMP-induced phosphorylation and allosteric conformational changes, AMPK monitors cellular energy status and phosphorylates downstream targets that coordinate nutrient sensing with growth, development, and metabolic homeostasis and protect against oxidative stress [27–29]. However, whether combinations of multiple biocontrol measures modulate AMPK signalling in insect pests and whether such modulation contributes to the durability of combined control remain unknown.

The brown planthopper (BPH) is one of the most destructive insect pests of rice in Asia. This pest causes severe crop losses through direct feeding on phloem sap, contamination of plants with honeydew, and transmission of plant viruses [30]. Current control strategies for BPH rely largely on insect-resistant rice varieties, chemical insecticides, and biological control agents. However, BPH exhibits high evolutionary potential and can rapidly develop new biotypes capable of overcoming major rice resistance genes [31]. In 1975, the International Rice Research Institute classified BPH populations into five biotypes and a *Leersia biotype* based on differences in virulence: populations unable to damage cultivars carrying resistance genes were designated biotype I; those able to damage cultivars carrying *Bph1* or *bph2* were designated biotypes II and III, respectively; those able to adapt to rice cultivars carrying *Bph3* or *bph4* were designated biotypes IV and V, respectively; and those damaging only *Leersia hexandra* but not rice were designated the Leersia biotype [32]. In addition, resistance to numerous active insecticide ingredients has been reported, with resistance documented for at least 33 compounds [33]. Alarmingly, cross-resistance between insecticide resistance and adaptation to resistant rice varieties has also been reported, further undermining the effectiveness of these control strategies [13, 34–36]. These challenges highlight the urgent need for more sustainable pest management approaches that reduce selection pressure for resistance while maintaining effective population suppression. EPF offers promising opportunities in this context. A specific strain of *Metarhizium anisopliae,* CQMa421, has recently been isolated and developed for commercial application. This strain has demonstrated effective control against several insect pests, including BPH, while showing minimal negative effects on natural enemies [37]. Despite these promising characteristics, the long-term compatibility and evolutionary consequences of integrating insect-resistant rice with this fungal pathogen remain poorly understood. In particular, it remains unclear whether combining host plant resistance with EPF could enhance pest suppression while reducing the rate of adaptive responses in BPH populations.

In the present study, we addressed these questions by examining the combined effects of insect-resistant rice and *M. anisopliae* on BPH populations across multiple generations. The integration of host plant resistance with fungal biocontrol would create complementary selection pressures that constrain pest adaptation and improve long-term control. Our results demonstrate an integrated and sustainable interaction between insect-resistant rice and *M. anisopliae*, with BPH populations remaining susceptible to both control strategies after 13 generations. Furthermore, mechanistic analyses reveal that this sustained susceptibility is associated with changes in insect energy metabolism, providing new insights into the physiological mechanisms underlying the durability of integrated pest management strategies. These findings contribute to the development of more sustainable pest control approaches that combine resistant crops with biological control agents, consistent with the goals of environmentally responsible and evolutionarily informed pest management.

## Results

### Compatibility between insect-resistant rice and *M. anisopliae*

Cross-resistance between host plant adaptation and chemical insecticide resistance is a major constraint limiting the effective integration of insect-resistant crops and insecticides in pest management programs [38]. To determine whether the adaptation of BPHs to the insect-resistant rice variety IR36 results in the development of resistance to commonly used insecticides, we evaluated insecticide susceptibility in the IR36-adapted population (P-AD) using the susceptible population (P-TN1) as a control.

A panel of insecticides commonly used for BPH control was tested, including both chemical insecticides and a microbial biocontrol agent. The results showed that the P-AD population, which had adapted to insect-resistant rice IR36, remained highly susceptible to the microbial insecticide *M. anisopliae* CQMa421. In contrast, this population exhibited increased resistance to several chemical insecticides, including pymetrozine, hexaflumuron, clothianidin, thiazophos, thiacloprid, and propyzamide. Resistance ratios ranged from 1.92- to 3.46-fold relative to the control population (Table 1; Table S2).

**Table 1:** Cross-resistance of the adapted population (P-AD) of BPHs to ***Metarhizium anisopliae*** CQMa421, pymetrozine, and thiamethoxam

**Insecticide**	**Population**	**LC**_**50**_** (95% F.L.)** **(mg/L or 10**^**8**^** conidia/mL)**	**Slope ± SE**	**χ**^**2**^** (df)**	**RR**^**a**^	**LCR**^**a**^
*M. anisopliae* CQMa421	P-TN1	1.05 (0.73–1.59)	0.93 ± 0.08	4.42 (3)	–	–
	P-AD	0.98 (0.35–5.14)	0.87 ± 0.08	9.11 (3)	0.93	1.07 (0.61–1.88)
Pymetrozine	P-TN1	52 (46–59)	2.71 ± 0.36	2.30 (3)	–	–
	P-AD	132 (110–167)	1.92 ± 0.34	2.11 (3)	2.54	0.39 (0.31–0.50) *
Thiamethoxam	P-TN1	6.50 (5.70–7.40)	2.48 ± 0.19	5.12 (3)	–	–
	P-AD	18 (14–23)	1.87 ± 0.17	0.72 (3)	2.69	0.37 (0.28–0.48) *

LCR (lethal concentration ratio) is the ratio of the LC50 of the adapted population (P-AD) to that of the control population (P-TN1); if this 95% confidence interval does not contain the value 1, the result is considered statistically significant at the 5% level and is marked with an asterisk (*). The LC_50_ of *M. anisopliae* CQMa421 is reported in units of 10^8^ conidia/mL, while LC_50_ values for chemical insecticides are reported in mg/L

**Fig. 1:**
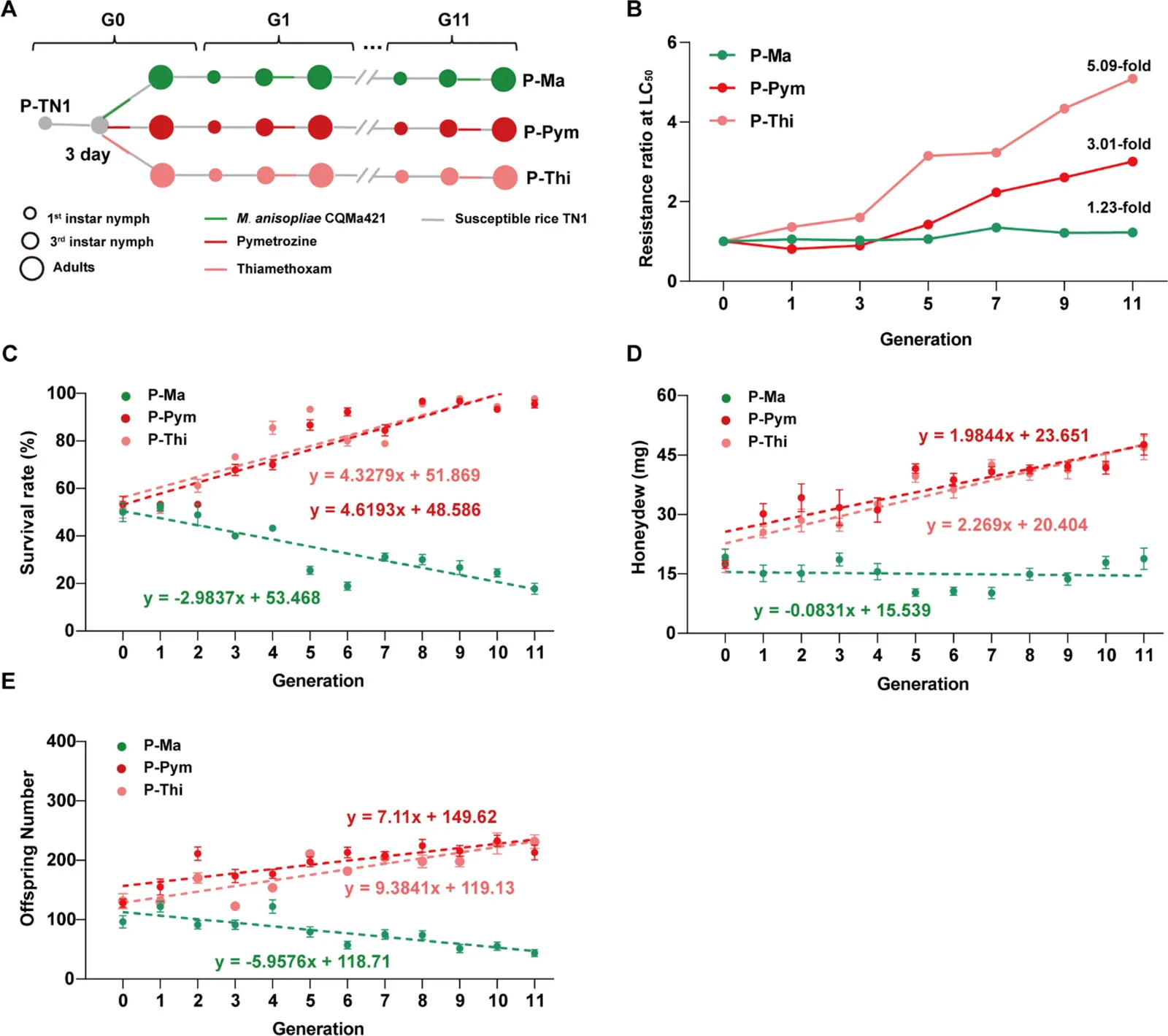
Multi-generational effects of sublethal exposure to *Metarhizium anisopliae* CQMa421, pymetrozine, and thiamethoxam on the brown planthopper (BPH) adaptation to insect-resistant rice IR36. **A** Experimental design for continuous selection across generations: 200 pairs of one-day-old female and male BPH adults were mated to produce the next generation. Third-instar nymphs were treated with sublethal concentrations (LC_10_) of *M. anisopliae* CQMa421, pymetrozine, or thiamethoxam. Survivors were reared to adulthood, and phenotypic assessments, including survival, honeydew excretion, and offspring number, were performed. This process was repeated continuously for 11 generations. **B** Changes in insecticide resistance following repeated exposure to low doses of *M. anisopliae* CQMa421, pymetrozine, and thiamethoxam across generations. Resistance ratios were calculated relative to the original P-TN1 population. **C**-**D** Adaptation of female adults to insect-resistant rice IR36 across generations, measured as survival rate, (*N* = 3 biological replicates, *n* = 30 individuals per replicate) honeydew excretion (*n* = 30 individuals), and total offspring production (*N* = 3 biological replicates, *n* = 50 pairs per replicate). Data represent the mean ± SEM of three biological replicates

Based on these results, one microbial insecticide (*M. anisopliae* CQMa421) and two representative chemical insecticides (thiamethoxam and pymetrozine) were selected for long-term exposure experiments. BPH populations were continuously exposed to sublethal concentrations (LC_10_) of these compounds across multiple generations to evaluate potential changes in insecticide resistance and host adaptation (Fig. 1A).

The results showed that the population exposed to *M. anisopliae* CQMa421 (P-Ma) did not exhibit any significant change in susceptibility after multiple generations of exposure, maintaining a sensitivity level comparable to that of the initial population (Fig. 1B). In contrast, populations exposed to chemical insecticides developed increased resistance over time. Specifically, resistance levels in the pymetrozine-exposed population (P-Pym) and the thiamethoxam-exposed population (P-Thi) increased by 3.0-fold and 5.1-fold, respectively, following prolonged exposure to sublethal doses (Fig. 1B).

To further assess whether these treatments influenced host adaptation, biological performance on insect-resistant rice IR36 was evaluated after 13 generations of exposure. The P-Ma population showed a significant decrease in the survival rate (*R*^2^ = 0.7273, *P* = 0.0004, *n* = 12) but no significant changes in honeydew excretion (*R*^2^ = 0.0914, *P* = 0.3366, *n* = 12) or offspring production (*R*^2^ = 0.5282, *P* = 0.0076, *n* = 12) on IR36 compared with the initial population, indicating that susceptibility to the resistant host plant remained stable (Fig. 1C-E).

In contrast, both the P-Pym and P-Thi populations exhibited significantly improved performance on IR36. These populations showed increased survival (P-Pym: *R*^2^ = 0.8370, *P* < 0.0001; P-Thi: *R*^2^ = 0.7528, *P* = 0.0003; *n* = 12 for both), higher honeydew excretion (P-Pym: *R*^2^ = 0.8597, *P* < 0.0001; P-Thi: *R*^2^ = 0.8460, *P* < 0.0001; *n* = 12 for both), and greater fecundity (P-Pym: *R*^2^ = 0.8370, *P* < 0.0001; P-Thi: *R*^2^ = 0.7528, *P* = 0.0003; *n* = 12 for both) when feeding on the resistant rice variety, indicating enhanced adaptation to the host plant (Fig. 1C-E).

Altogether, these findings demonstrate that exposure to chemical insecticides can promote both insecticide resistance and increased adaptation to resistant host plants. In contrast, the microbial insecticide *M. anisopliae* CQMa421 did not induce similar adaptive responses. These results highlight the higher compatibility of insect-resistant rice IR36 with *M. anisopliae* CQMa421 compared with conventional chemical insecticides, suggesting that combining resistant crops with microbial biocontrol agents may represent a more sustainable strategy for the long-term management of BPH populations.

### The combination of insect-resistant rice and *M. anisopliae* provides prolonged control efficacy against BPH

**Fig. 2:**
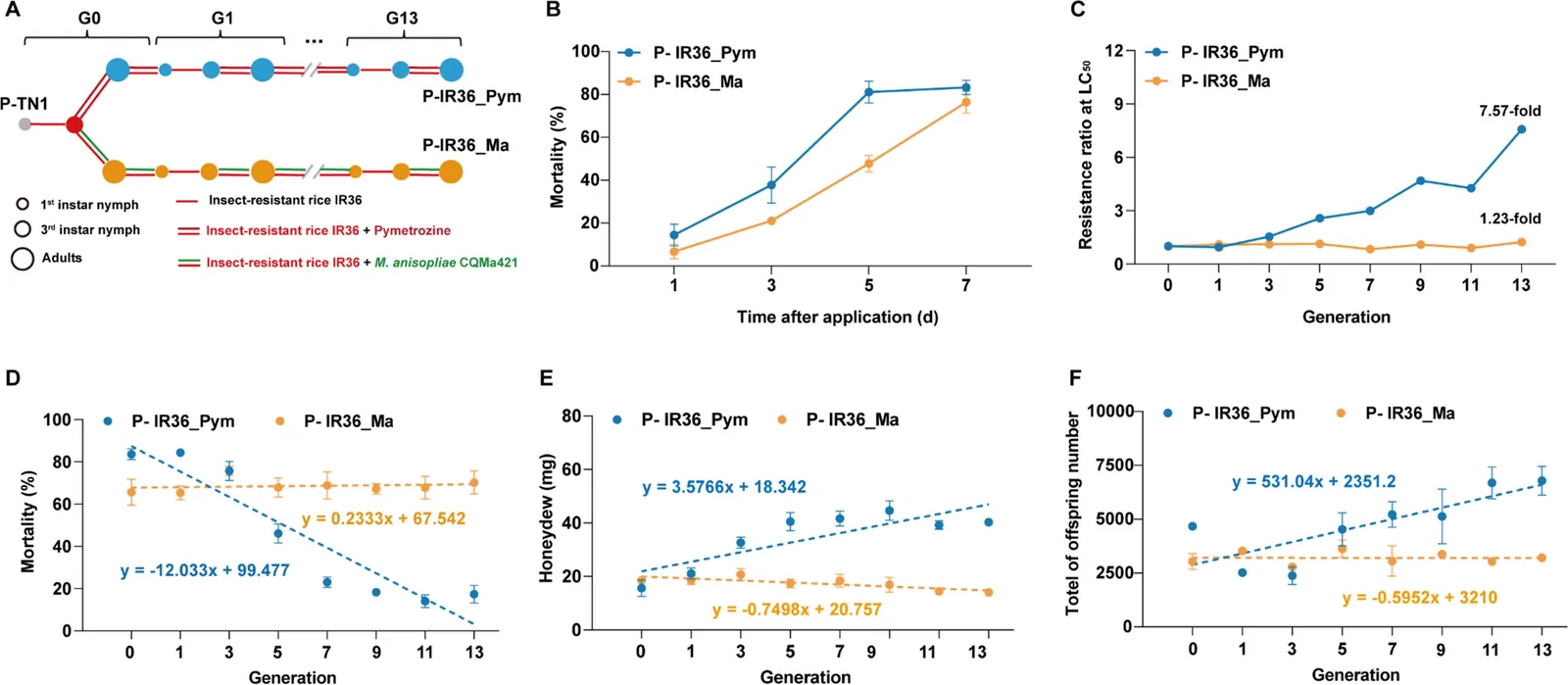
Integrated effects of insect-resistant rice IR36 and *M. anisopliae* CQMa421 on sustainable BPH management. **A** Experimental design for multi-generational assessment: BPHs were continuously reared on insect-resistant rice IR36, treated at the third-instar stage with *M. anisopliae* CQMa421 or pymetrozine, and phenotypically assessed as adults. This cycle was repeated for 13 generations. **B** Short-term integrated effects of combining IR36 with *M. anisopliae* CQMa421 or pymetrozine, showing mortality over 7 days. **C**-**D** Long-term impacts of continuous combined treatments on BPH resistance and control efficacy. Resistance ratios and mortality were monitored across generations, highlighting that chemical insecticide exposure promotes resistance development, whereas *M. anisopliae* CQMa421 maintains sustained susceptibility. Mortality was assessed in three biological replicates, each comprising 30 individuals. **E**–**F** Ecological fitness of BPHs on IR36 under different combined treatments, including honeydew excretion (*n* = 30 individuals) and total offspring production (*N* = 3 biological replicates, *n* = 3, 50 pairs per replicate). The results demonstrate that the integration of IR36 with *M. anisopliae* CQMa421 sustains control efficacy while limiting adaptive fitness increases, supporting a durable and environmentally friendly pest management strategy

We evaluated the efficacy of combining the insect-resistant rice variety IR36 with different insecticides for the control of BPHs (Fig. 2A). The results showed that when IR36 was combined with the chemical insecticide pymetrozine, a mortality rate of 83% was achieved within five days (Fig. 2B). In comparison, the combination of IR36 with the microbial insecticide *M. anisopliae* CQMa421 resulted in a mortality rate of 76% after seven days (Fig. 2B). These findings indicate that both combinations can substantially enhance pest suppression in the short term, although the microbial agent exhibited a slightly slower but still effective action.

To further investigate whether these control strategies remain effective over time, we examined the long-term effects of combining resistant rice with different insecticides across multiple generations. After 13 generations of treatment with pymetrozine, the population P-IR36_Pym exhibited a marked increase in resistance, reaching 7.6-fold relative to the initial population (Fig. 2C). In contrast, the population P-IR36_Ma, which was exposed to *M. anisopliae* CQMa421 in combination with IR36, showed no evidence of resistance development. Instead, the susceptibility of this population remained stable, with a resistance ratio of only 0.69-fold compared with the original population (Fig. 2C). These results suggest that the integration of resistant rice with pymetrozine promotes resistance evolution, whereas the combination with *M. anisopliae* CQMa421 does not significantly drive resistance development.

Consistent with this pattern, the long-term control efficacy of the two strategies differed substantially. After 13 generations of treatment, the effectiveness of the IR36-pymetrozine combination declined dramatically, with mortality decreasing from 84 to 14% (*R*^2^ = 0.8889, *P* = 0.0008, *n* = 8). In contrast, the control efficacy of the IR36-*M. anisopliae* CQMa421 combination remained stable over time, with BPH mortality consistently ranging between 65 and 76% (Fig. 2D, R^2^ = 0.0065, *P* = 0.8504, *n* = 8). These results demonstrate that the microbial-based strategy provides more durable pest suppression than the chemical insecticide when combined with resistant rice.

To further understand the biological basis of these differences, we evaluated the fitness of the P-IR36_Pym and P-IR36_Ma populations when feeding on IR36. The P-IR36_Pym population exhibited a clear increase in fitness, characterized by enhanced feeding activity and reproductive performance. Specifically, honeydew production increased significantly (Fig. 2E R^2^ = 0.7861, *P* = 0.0031, *n* = 8), and the total number of offspring produced by 50 mating pairs rose from 4671 to 6789 after 13 generations, representing a 1.5-fold increase (Fig. 2F, R^2^ = 0.7933, *P* = 0.0029, *n* = 8). These results indicate that prolonged exposure to pymetrozine not only promoted insecticide resistance but also enhanced the ability of BPHs to exploit the resistant host plant.

In contrast, the P-IR36_Ma population maintained consistently low feeding activity and reproductive output when reared on IR36. Honeydew production remained relatively stable throughout the experiment, ranging from 13.99 to 18.61 mg (Fig. 2E), suggesting sustained sensitivity to the resistant rice. Similarly, the total number of offspring produced by 50 mating pairs remained stable, varying between 2787 and 3367 across generations (Fig. 2F). These results indicate that long-term exposure to *M. anisopliae* CQMa421 did not promote adaptive responses that enhance BPH performance in resistant rice.

These findings demonstrate that although both chemical and microbial insecticides can enhance the short-term effectiveness of insect-resistant rice, their long-term outcomes differ markedly. While the combination with chemical insecticides accelerates resistance evolution and host adaptation, the integration of resistant rice with the microbial insecticide *M. anisopliae* CQMa421 provides more durable and sustainable pest suppression. It should be noted that the transgenerational effects described here refer to the maintenance of susceptibility across multiple generations through continued exposure to *M. anisopliae* CQMa421, rather than to vertical transmission of conidia or direct effects on untreated offspring.

### Combination of insect-resistant rice and *M. anisopliae* induces systemic lipid redistribution in BPHs

Insects activate diverse defense mechanisms under stress conditions, which require substantial energy expenditure, with lipid mobilization playing a central role [39]. To investigate how energy metabolism responds to combined exposure to insect-resistant rice IR36 and different insecticides, we compared lipid profiles and energy metabolites in BPH populations P-IR36_Pym and P-IR36_Ma. Specifically, we measured ATP as a direct source of energy and TAG and DAG as key forms of energy storage and mobilization.

**Fig. 3:**
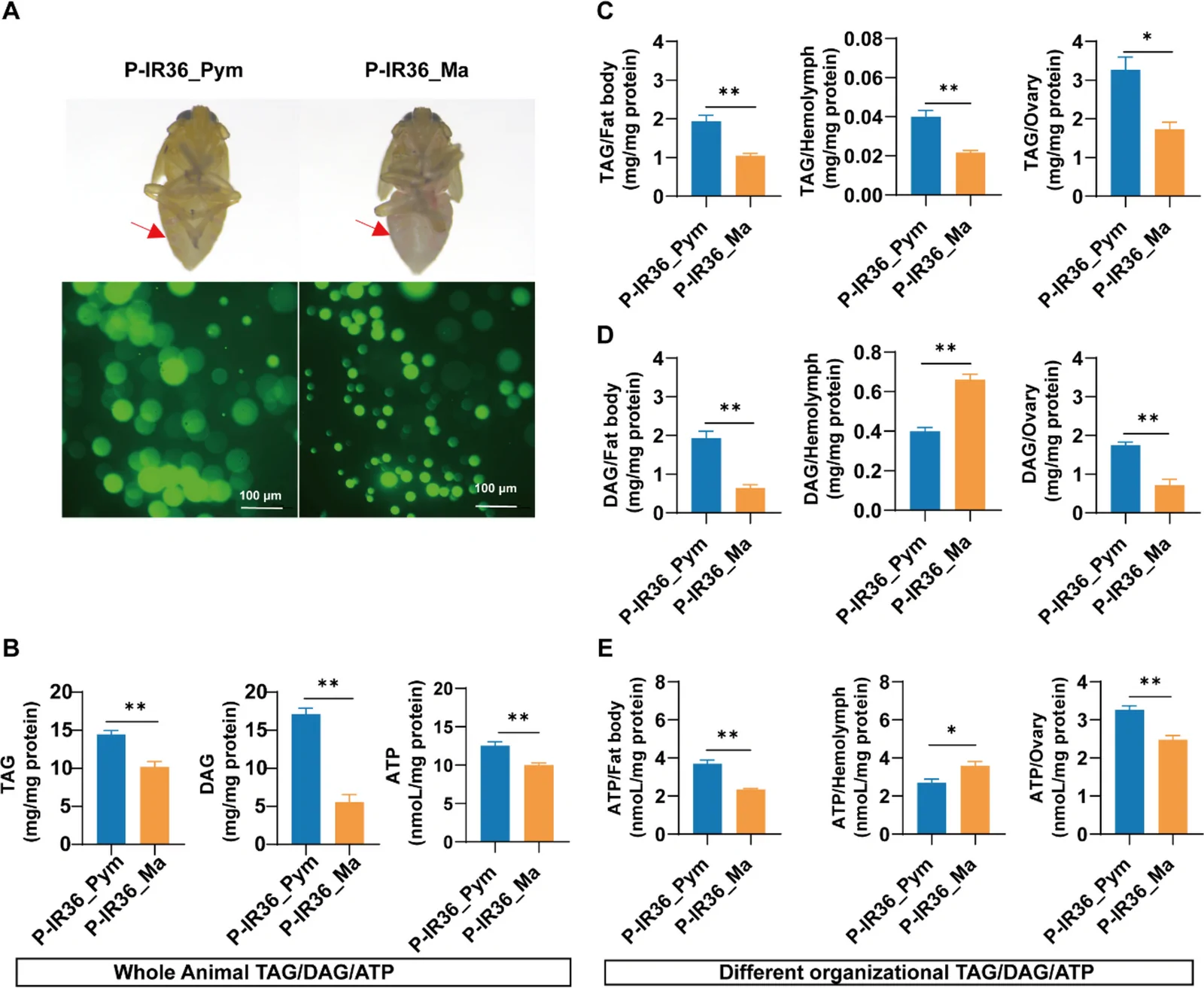
Lipid metabolism in BPHs under the combined effect of insect-resistant rice IR36 and different insecticides. **A** Visualization of neutral lipids in the epidermis and lipid droplets in fat body tissues using Eosin Y and BODIPY staining, revealing differences in lipid content and droplet morphology between treatments. **B** Quantification of energy storage and mobilization metabolites, namely, triacylglycerol (TAG), diacylglycerol (DAG), and adenosine triphosphate (ATP), in the P-IR36_Pym and P-IR36_Ma populations (*n* = 3 biological replicates, 30 insects per replicate). **C**-**E** Tissue-specific distribution of TAG, DAG, and ATP across the fat body, hemolymph, and ovary in the two populations (*n* = 3 replicates, 150 insects per replicate), highlighting the redistribution of mobilizable lipids toward hemolymph under the combined stress of resistant rice and *M. anisopliae* CQMa421. Data are presented as means ± SEM; * *P* < 0.05, ** *P* < 0.01 (Student’s t-test). P-IR36_Pym: population reared on IR36 and treated with pymetrozine; P-IR36_Ma: population reared on IR36 and treated with *M. anisopliae* CQMa421

Our analyses revealed distinct metabolic responses between the populations. The P-IR36_Ma population exhibited significantly lower TAG, DAG and ATP levels in whole-body measurements than P-IR36_Pym (Fig. 3A, B). Consistent with these biochemical changes, lipid droplet staining of fat body tissue showed that the P-IR36_Ma population contained fewer lipid droplets and displayed greater variability in droplet size (Fig. 3A). These findings suggest that exposure to IR36 in combination with *M. anisopliae CQMa421* suppresses energy synthesis while inhibiting lipid degradation and mobilization in BPHs.

To further understand the tissue-specific dynamics of lipid metabolism, we measured energy metabolite levels in the fat body, hemolymph, and ovary, which are key tissues involved in detoxification, immune defense, and reproduction, respectively [40]. Across all examined tissues, TAG levels were significantly reduced in the P-IR36_Ma population compared with P-IR36_Pym (Fig. 3C). DAG levels decreased in the fat body and ovary but were significantly elevated in the hemolymph (Fig. 3D), suggesting a systemic redistribution of mobilizable lipids. ATP levels followed a trend similar to that of DAG in the fat body and ovary, being significantly reduced in P-IR36_Ma, whereas hemolymph ATP was significantly elevated (Fig. 3E), indicating enhanced energy availability in hemolymph under these conditions.

### The combination of insect-resistant rice and *M. anisopliae* activates AMPK to drive lipid mobilization

**Fig. 4:**
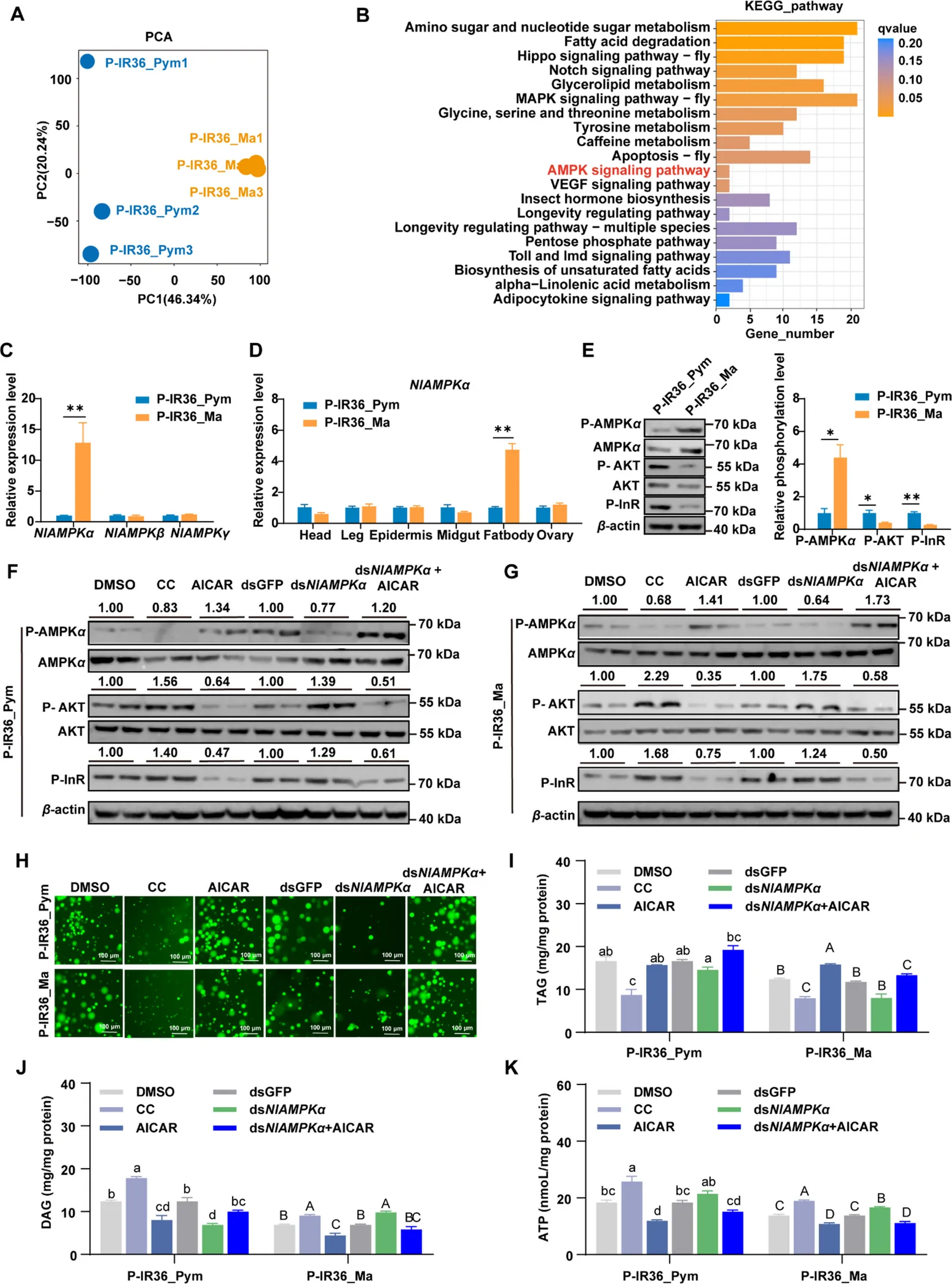
Integrated application of insect-resistant rice IR36 and *M. anisopliae* CQMa421 activates AMPK in the fat body, suppressing insulin signaling and modulating lipid metabolism in BPHs. **A**-**B** Principal component analysis (PCA) and KEGG pathway enrichment of differentially expressed genes in the P-IR36_Pym and P-IR36_Ma populations, highlighting energy metabolism-related pathways, including AMPK signaling. **C**-**D** Relative expression levels of *NlAMPKα*, *NlAMPKβ*, and *NlAMPKγ* between populations, with tissue-specific profiling revealing high *NlAMPKα* expression in the fat body of P-IR36_Ma. **E** Western blot analysis showing phosphorylation levels of AMPKα and insulin signaling pathway proteins (InR and AKT) in the fat body, demonstrating an inverse correlation between AMPK activation and IIS. Data are means ± SEM; * *P* < 0.05, ** *P* < 0.01 (Student’s *t*-test). **F**-**K** Effects of pharmacological and RNAi manipulation of AMPK activity on insulin signaling, fat body lipid droplet morphology, and whole-insect energy metabolites (TAG, DAG, ATP). Treatments included DMSO (control), Compound C (CC; AMPK inhibitor), AICAR (AMPK activator), dsGFP (RNAi control), ds*NlAMPKα* (AMPKα knockdown) and ds*NlAMPKα* + AICAR (rescue assay). Numbers above the bands in **F** and **G** indicate the relative expression levels of phosphorylated proteins, normalized to 1.00 for the DMSO group in pharmacological experiments and the dsGFP group in RNAi and rescue assays. Data are means ± SEM; uppercase and lowercase letters denote metabolite differences among different brown planthopper populations under different treatments, one-way ANOVA followed by Tukey's HSD test (*P* < 0.05). P-IR36_Pym: population reared on IR36 and treated with pymetrozine; P-IR36_Ma: population reared on IR36 and treated with *M. anisopliae* CQMa421

To elucidate the mechanisms underlying systemic lipid redistribution in BPHs under the combined influence of insect-resistant rice IR36 and *M. anisopliae* CQMa421, we performed transcriptome sequencing on one-day-old female adults from the 13th generation of the P-IR36_Pym and P-IR36_Ma populations. Comparative analysis revealed significant differences in gene expression between the two populations (Fig. 4A). KEGG pathway enrichment analysis of differentially expressed genes demonstrated that these genes were predominantly associated with energy-related pathways, including fatty acid degradation, amino sugar and nucleotide sugar metabolism, glycerol metabolism, Hippo signaling, Notch signaling, MAPK signaling, tyrosine signaling, and notably, the AMPK signaling pathway (Fig. 4B). Given the central role of AMPK as a cellular energy sensor coordinating metabolism and energy expenditure [41], we focused on its potential function in regulating lipid redistribution in BPHs.

RT-qPCR revealed differential expression among AMPK subunits in the two populations. Specifically, *NlAMPKα* was highly expressed in the P-IR36_Ma population, whereas *NlAMPKβ* and *NlAMPKγ* showed no significant differences (Fig. 4C). Spatiotemporal expression analysis further demonstrated that *NlAMPKα* expression was particularly elevated in the fat body of P-IR36_Ma insects (Fig. 4D), suggesting a key role for this subunit in lipid metabolic regulation.

In model insects such as *Drosophila* and *Bombyx*, AMPK phosphorylation has been shown to inhibit the insulin/insulin-like signaling (IIS) pathway [42]. To test whether this mechanism applies to BPHs, we measured the phosphorylation levels of AMPKα, insulin receptor (InR), and protein kinase B (AKT) in fat body tissues from the two populations. Western blot analysis revealed that P-IR36_Ma individuals exhibited elevated AMPKα phosphorylation, accompanied by reduced phosphorylation of InR and AKT. Conversely, P-IR36_Pym insects showed lower AMPKα phosphorylation with higher InR and AKT phosphorylation (Fig. 4E), indicating a potential negative correlation between AMPK activation and IIS in the fat body.

To validate the regulatory relationship between AMPK and IIS, we conducted pharmacological and RNAi experiments. Activation of AMPK with 5-aminoimidazole − 4-carboxamide ribonucleotide (AICAR) in one-day-old female adults from both populations enhanced AMPKα phosphorylation and suppressed InR and AKT phosphorylation in fat body tissues (Fig. 4F, G). In contrast, inhibition of AMPK using Compound C (an ATP-competitive inhibitor) or ds*NlAMPKα* RNAi reduced AMPKα phosphorylation and increased InR and AKT phosphorylation (Fig. 4F, G). Notably, the rescue assay of ds*NlAMPKα* + AICAR reversed the effects of ds*NlAMPKα* alone, restoring AMPKα phosphorylation and suppressing InR and AKT phosphorylation to levels comparable to AICAR treatment alone (Fig. 4F, G). These results confirm that AMPK negatively regulates the IIS pathway in BPH fat bodies.

To examine the functional consequences of AMPK-mediated IIS suppression on lipid metabolism, we assessed lipid droplet and energy metabolite levels following pharmacological and RNAi treatments. Activation of AMPK via AICAR increased the number of lipid droplets in fat body tissue (Fig. 4H), increased total TAG content, and simultaneously decreased DAG and ATP levels within the whole insect (Fig. 4I–K). Conversely, inhibition of AMPK using Compound C or ds*NlAMPKα* resulted in decreased lipid droplet accumulation, reduced TAG content, and enhanced DAG and ATP levels (Fig. 4H–K). Importantly, the ds*NlAMPKα* + AICAR combination treatment partially rescued the ds*NlAMPKα*-induced phenotype: lipid droplet numbers were increased compared with ds*NlAMPKα* alone (Fig. 4H), TAG content was significantly increased (Fig. 4I), and DAG and ATP levels were reduced (Fig. 4J, K), although in some cases, these values did not fully return to AICAR-only levels. These results indicate that AMPK regulates lipid mobilization in BPHs by modulating IIS in the fat body, promoting the conversion of storage lipids into mobilizable forms to support energy demands under the stress of resistant rice and microbial biocontrol.

### AMPK-mediated lipid redistribution is essential for maintaining BPH sensitivity to insect-resistant rice and *M. anisopliae*

**Fig. 5:**
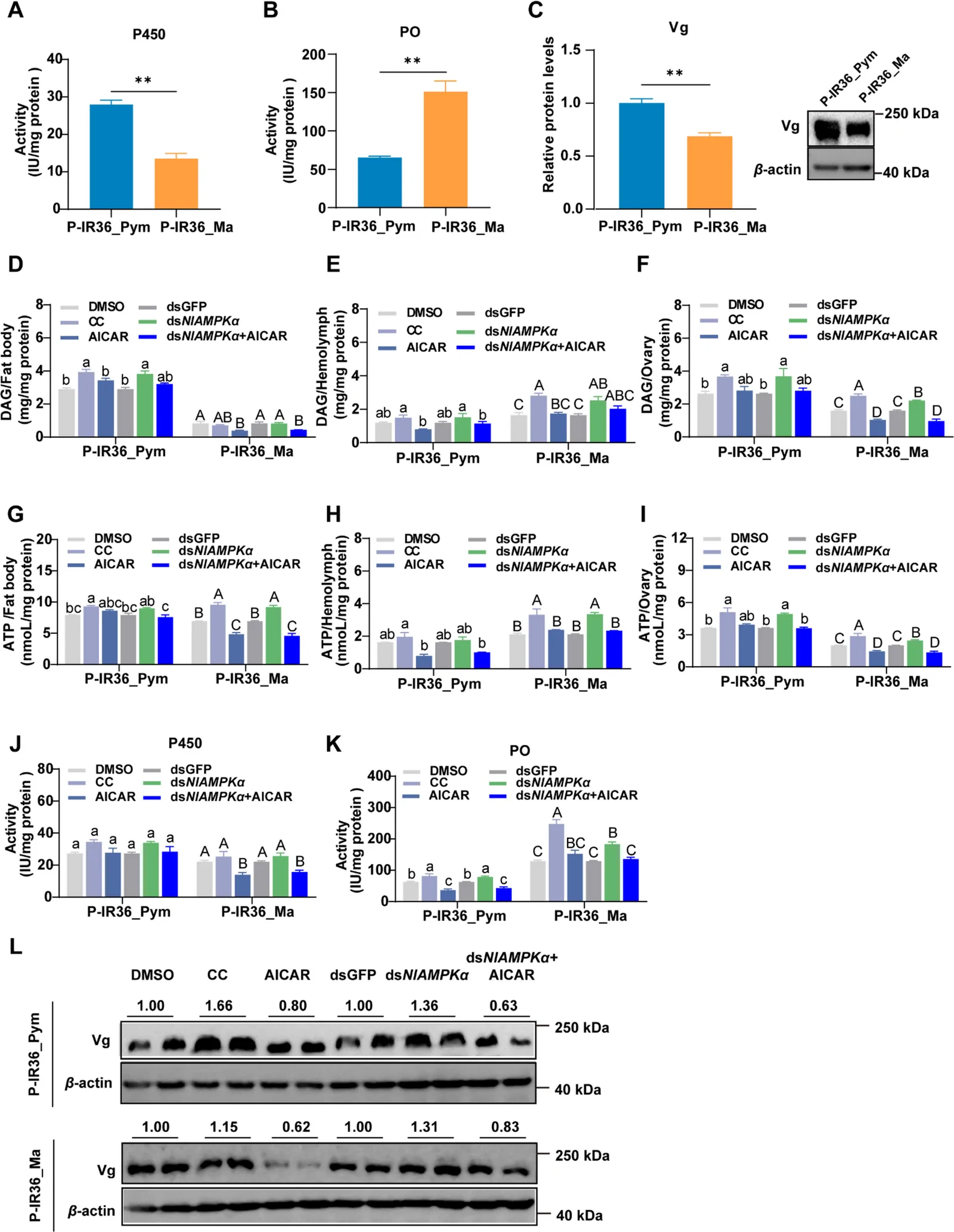
AMPK regulates tissue-specific energy substrate reallocation and enzyme activity in BPHs under combined stress from insect-resistant rice IR36 and *M. anisopliae* CQMa421. **A**-**C** Baseline activities of detoxification and metabolic enzymes, including cytochrome P450 (P450) in the fat body, polyphenol oxidase (PO) in the hemolymph, and vitellogenin (Vg) protein expression in the ovary, comparing the P-IR36_Pym and P-IR36_Ma populations. Data are presented as means ± SEM; * *P* < 0.05, ** *P* < 0.01 (Student’s *t*-test). **D**-**F** Effects of pharmacological and RNAi modulation of AMPK activity on DAG levels across the fat body, hemolymph, and ovary, showing enhanced lipid mobilization toward hemolymph under elevated AMPK activity. **G**-**I** Tissue-specific changes in ATP content following AMPK activation or inhibition, reflecting energy redistribution across key organs. **J**-**L** Corresponding alterations in enzymatic activities (P450, PO) and Vg protein expression after AMPK manipulation, demonstrating the linkage between energy allocation and metabolic/immune/reproductive functions. Numbers above the bands in Fig. 5 L represent the relative expression ratios of the Vg proteins to *β*-actin. normalized to 1.00 for the DMSO group in pharmacological experiments and the dsGFP group in RNAi and rescue assays. Data are means ± SEM; uppercase and lowercase letters denote metabolite differences among different brown planthopper populations under different treatments, one-way ANOVA followed by Tukey's HSD test (*P* < 0.05). P-IR36_Pym: population reared on IR36 and treated with pymetrozine; P-IR36_Ma: population reared on IR36 and treated with *M. anisopliae* CQMa421

To evaluate the functional significance of lipid redistribution in BPHs under combined stress from insect-resistant rice IR36 and *M. anisopliae*, we first assessed key metabolic and reproductive markers in the P-IR36_Pym and P-IR36_Ma populations. Compared with P-IR36_Pym, the P-IR36_Ma population exhibited lower cytochrome P450 activity in the fat body and reduced vitellogenin (Vg) protein expression in the ovary, while polyphenol oxidase (PO) activity in the hemolymph was elevated (Figs. 5A-C). These patterns were consistent with tissue-specific energy metabolite levels previously observed (Fig. 3D, E), suggesting a positive correlation between DAG/ATP concentrations and the activity of detoxification, immunity, and reproductive enzymes.

To directly test the role of AMPK in regulating tissue-specific lipid allocation, we performed pharmacological and RNAi experiments. Activation of AMPK with AICAR in P-IR36_Ma insects led to a significant decrease in DAG levels in the fat body and ovary, accompanied by an increase in the hemolymph. Conversely, the P-IR36_Pym population showed an increase in DAG levels within the fat body and ovary under the same treatment (Fig. 5D-F). Inhibition of AMPK using Compound C or ds*NlAMPKα* resulted in increased DAG levels (Fig. 5D-F). Notably, the combined treatment of ds*NlAMPKα* + AICAR partially reversed the ds*NlAMPKα*-induced changes: in P-IR36_Ma, DAG levels in the fat body and ovary were reduced compared with ds*NlAMPKα* alone, while hemolymph DAG was elevated (Fig. 5D-F). ATP levels across tissues exhibited similar trends to DAG, further indicating that AMPK regulates energy mobilization systemically (Fig. 5G-I), with ds*NlAMPKα* + AICAR treatment restoring ATP levels toward those observed under AICAR alone in several tissues.

Assessment of enzyme activities and protein expression following AMPK modulation revealed population-specific responses. In P-IR36_Ma, the activation of AMPK led to increased PO activity and decreased P450 activity and Vg expression. In contrast, the P-IR36_Pym population displayed opposite trends, consistent with shifts in DAG and ATP metabolism (Fig. 5J-L). Importantly, the ds*NlAMPKα* + AICAR combination treatment in P-IR36_Ma resulted in intermediate phenotypes: PO activity was elevated but lower than with ds*NlAMPKα* alone, while P450 activity and Vg expression were partially restored compared with ds*NlAMPKα* alone (Fig. 5J-L). These findings indicate that AMPK-mediated energy redistribution modulates the balance between detoxification, immunity, and reproduction in a population-specific manner.

**Fig. 6:**
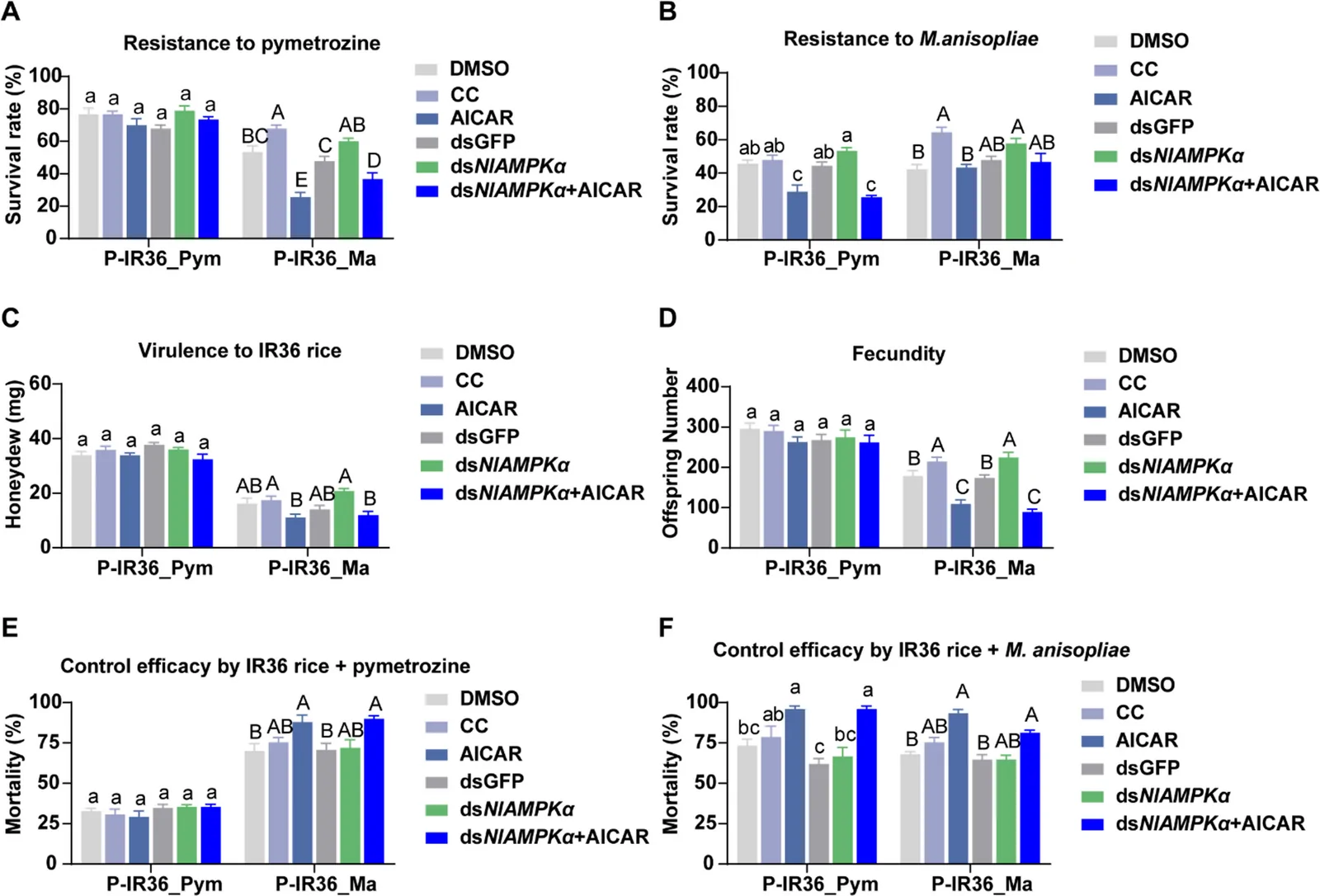
AMPK activity modulates BPH fitness and influences the efficacy of integrated pest management combining insect-resistant rice IR36 with chemical and microbial insecticides. **A**-**D** Effects of pharmacological and RNAi manipulation of AMPK activity on key fitness traits of P-IR36_Pym and P-IR36_Ma populations, including resistance to pymetrozine (**A**), susceptibility to *M. anisopliae* CQMa421 (**B**), feeding virulence on insect-resistant rice IR36 (**C**), and reproductive output (**D**). Changes in these traits reflect the role of AMPK in regulating energy allocation between detoxification, immunity, and reproduction. **E**–**F** Assessment of the long-term control effectiveness of integrated pest management strategies after modulation of AMPK activity, evaluating the combined application of insect-resistant rice IR36 with either *M. anisopliae* CQMa421 or chemical insecticides. Data are means ± SEM; uppercase and lowercase letters denote metabolite differences among different brown planthopper populations under different treatments, one-way ANOVA followed by Tukey's HSD test (*P* < 0.05). P-IR36_Pym: population reared on IR36 and treated with pymetrozine; P-IR36_Ma: population reared on IR36 and treated with *M. anisopliae* CQMa421

Functional bioassays further confirmed the significance of these metabolic changes. Modulation of AMPK activity in P-IR36_Pym did not alter resistance to pymetrozine, performance on IR36, or reproductive output, while the population remained highly sensitive to *M. anisopliae* CQMa421 (Fig. 6A–D). In contrast, activated AMPK in P-IR36_Ma significantly reduced chemical resistance, virulence on IR36, and reproductive capacity, while sensitivity to *M. anisopliae* remained high (Fig. 6A–D). Notably, the ds*NlAMPKα* + AICAR combination treatment partially reversed the ds*NlAMPKα*-induced phenotypes in P-IR36_Ma: the survival rate on pymetrozine was restored to a level comparable to AICAR alone (Fig. 6A), the survival rate on *M. anisopliae* was reduced relative to ds*NlAMPKα* alone (Fig. 6B), honeydew excretion on IR36 was decreased (Fig. 6C), and fecundity was markedly reduced compared with ds*NlAMPKα* alone (Fig. 6D). These results suggest that BPHs deploy distinct energy allocation strategies under different stressors and that AMPK-mediated lipid redistribution supports sustained susceptibility under microbial biocontrol and resistant rice pressure.

Finally, to directly link AMPK-mediated lipid mobilization to pest control efficacy, we examined the impact of AMPK activation and inhibition on mortality under combined treatments of IR36 and pymetrozine or *M. anisopliae*. Treatment with AICAR or the combined treatment of ds*NlAMPKα* + AICAR significantly enhanced the effectiveness of IR36 combined with pymetrozine in the P-IR36_Ma population (Fig. 6E). Notably, in the *M. anisopliae* combination treatment, treatment with AICAR or the combined treatment of ds*NlAMPKα* + AICAR all significantly enhanced control efficacy in both populations (Fig. 6F). These findings demonstrate that AMPK activity and the resulting lipid redistribution are key determinants of BPH sensitivity to integrated plant–microbe pest management strategies.

## Discussion

Reports of practical resistance to living microbial insecticides remain rare in field settings. For instance, *D. melanogaster* showed no change in resistance after 15 generations of exposure to *Beauveria bassiana* [43], and *Galleria mellonella* subjected to 25 generations of selection by *M. anisopliae* exhibited only minor fluctuations in mortality without evidence of resistance evolution [44]. Multi-generational exposure of BPHs to *M. anisopliae* CQMa421 did not induce measurable resistance under laboratory conditions in this study. The infection of insect pests by living microbial insecticides is a complex multistage process, beginning with conidial attachment and germination on the cuticle, followed by penetration, proliferation within the hemocoel, and ultimately host mortality [21]. In contrast to chemical insecticides, insect adaptation to microbial pathogens is typically polygenic, making resistance evolution slower and less likely [45, 46]. Here, adaptation to resistant host plants did not confer cross-resistance to *M. anisopliae* (Table 1), demonstrating that singular host adaptation or resistance genes do not compromise microbial efficacy.

Importantly, *M. anisopliae* CQMa421 also exhibited transgenerational control effects under laboratory conditions. Our findings show that after multiple generations of exposure, BPH survival on insect-resistant rice declined, suggesting that microbial biocontrol can influence subsequent generations. Previous studies report vertical transmission of conidia during insect mating, leading to reduced fecundity and increased offspring mortality [47–49]. Similarly, *M. anisopliae* Ma421-infected female *Anopheles sinensis* produced fewer eggs with lower hatching rates [50], reinforcing the potential of microbial biopesticides for sustained, multi-generational pest suppression. This transgenerational efficacy is particularly relevant for long-term integrated pest management (IPM), where preventing pest resurgence is critical.

The compatibility of living microbial insecticides with insect-resistant crops presents a promising avenue for sustainable pest control [51]. Microbial biopesticides are characterized by a low likelihood of resistance evolution, high host specificity, and environmental safety [52]. While integrated effects between microbial and chemical insecticides have been documented [53–55], practical application is limited. Broad-spectrum chemical insecticides often impair microbial biocontrol by inhibiting spore germination, hyphal growth, and infectivity [56–58]. Improper dosing or timing can trigger pest resurgence, and environmental factors such as temperature, humidity, and light further constrain efficacy [59–62]. In contrast, combining microbial biocontrol with insect-resistant crops avoids these pitfalls. Our results indicate that the integration of *M. anisopliae* CQMa421 with IR36 rice enhances control efficacy, suppresses host adaptation, and maintains the long-term susceptibility of BPHs, providing a durable, low-risk IPM strategy.

From a mechanistic perspective, energy metabolism plays a central role in BPH resilience. Insects face trade-offs between growth, reproduction, and defense, with immune responses imposing substantial energetic costs [15, 63–65]. Under dual stress from insect-resistant rice and microbial infection, BPHs must dynamically mobilize stored energy, converting TAG into DAG, which can also act as a lipid second messenger for systemic energy allocation [66–68]. Combined IR36 and *M. anisopliae* exposure elevates DAG levels while reducing ATP content. Tissue-specific analyses revealed that DAG decreased in the fat body and ovary but accumulated in the hemolymph, suggesting a preferential allocation of energy to support humoral immunity against microbial infection. This redistribution reflects an adaptive strategy where BPHs prioritize survival under biotic stress, although the available energy remains insufficient to overcome the combined pressure from resistant rice and microbial biocontrol. Although the tissue-specific patterns indicate a correlation between the availability of DAG/ATP in the hemolymph and detoxification (P450), immunity (PO), and reproduction (Vg), the direct regulatory relationships among them remain to be further verified.

Central to this redistribution is AMP-activated protein kinase (AMPK), a critical cellular energy sensor composed of *α*, *β*, and *γ* subunits that coordinates catabolic and anabolic pathways [69–72]. In insects, AMPK activation promotes catabolism, while insulin/insulin-like signaling (IIS) mediates anabolic processes, establishing a natural inhibitory relationship [42]. This study shows that elevated AMPKα phosphorylation suppresses the IIS signaling pathway, thereby inhibiting lipid mobilization. The fat body, functioning analogously to the mammalian liver, integrates nutrient sensing and energy storage while regulating development and immune responses [40]. By modulating IIS, AMPK enables systemic energy reallocation under stress, supporting humoral immunity while limiting resources available for growth and reproduction, thereby maintaining sensitivity to both resistant rice and microbial biocontrol agents. The functional consequences of these metabolic adjustments were further validated. Activation of AMPK reduced BPH virulence on IR36 and enhanced susceptibility to *M. anisopliae*. Conversely, inhibition of AMPK elevated resistance to chemical insecticides and host plant adaptation, demonstrating that AMPK-mediated lipid mobilization underlies long-term susceptibility to integrated plant–microbe pest control strategies. These findings provide a mechanistic explanation for the transgenerational and sustained efficacy of the IR36-*M. anisopliae* combination.

We also remark that, despite these insights, several knowledge gaps remain. First, while our study highlights the role of AMPK and IIS in energy allocation and pest susceptibility, the contributions of other signaling pathways, such as TOR, MAPK, and JAK/STAT, in coordinating immune-metabolic trade-offs under combined stress remain unclear [70, 73, 74]. Second, the long-term field performance of the IR36–*M. anisopliae* combination under variable environmental conditions, including different insect-resistant rice varieties carrying other *Bph* resistance genes, such as *Bph3*, *Bph14*, and *Bph15*, as well as temperature, humidity, and natural enemy presence, warrants further investigation. Third, while transgenerational effects of microbial biocontrol are promising, the genetic and epigenetic mechanisms driving these effects in BPHs remain to be elucidated. Our multi-generational selection used a single line per treatment; we therefore cannot fully exclude contributions from genetic drift, founder, bottleneck effects or lineage-specific variation to the observed population-level trajectories. Finally, the applicability of this integrated strategy to other major rice pests or in diverse cropping systems remains to be tested, which is critical for broad-scale implementation.

In terms of applied pest management, we believe that our findings provide strong evidence supporting the integration of resistant crops and microbial biocontrol to achieve sustainable pest suppression. By combining IR36 rice with *M. anisopliae* CQMa421, growers can leverage integrated effects that limit host adaptation, maintain susceptibility, and reduce reliance on chemical insecticides. This approach aligns with the principles of IPM, reducing environmental contamination, conserving natural enemies, and mitigating the evolution of resistance in pest populations. Furthermore, elucidating the molecular mechanisms of AMPK-mediated lipid redistribution provides a conceptual framework for identifying new targets to enhance the efficacy and durability of biocontrol strategies.

**Fig. 7:**
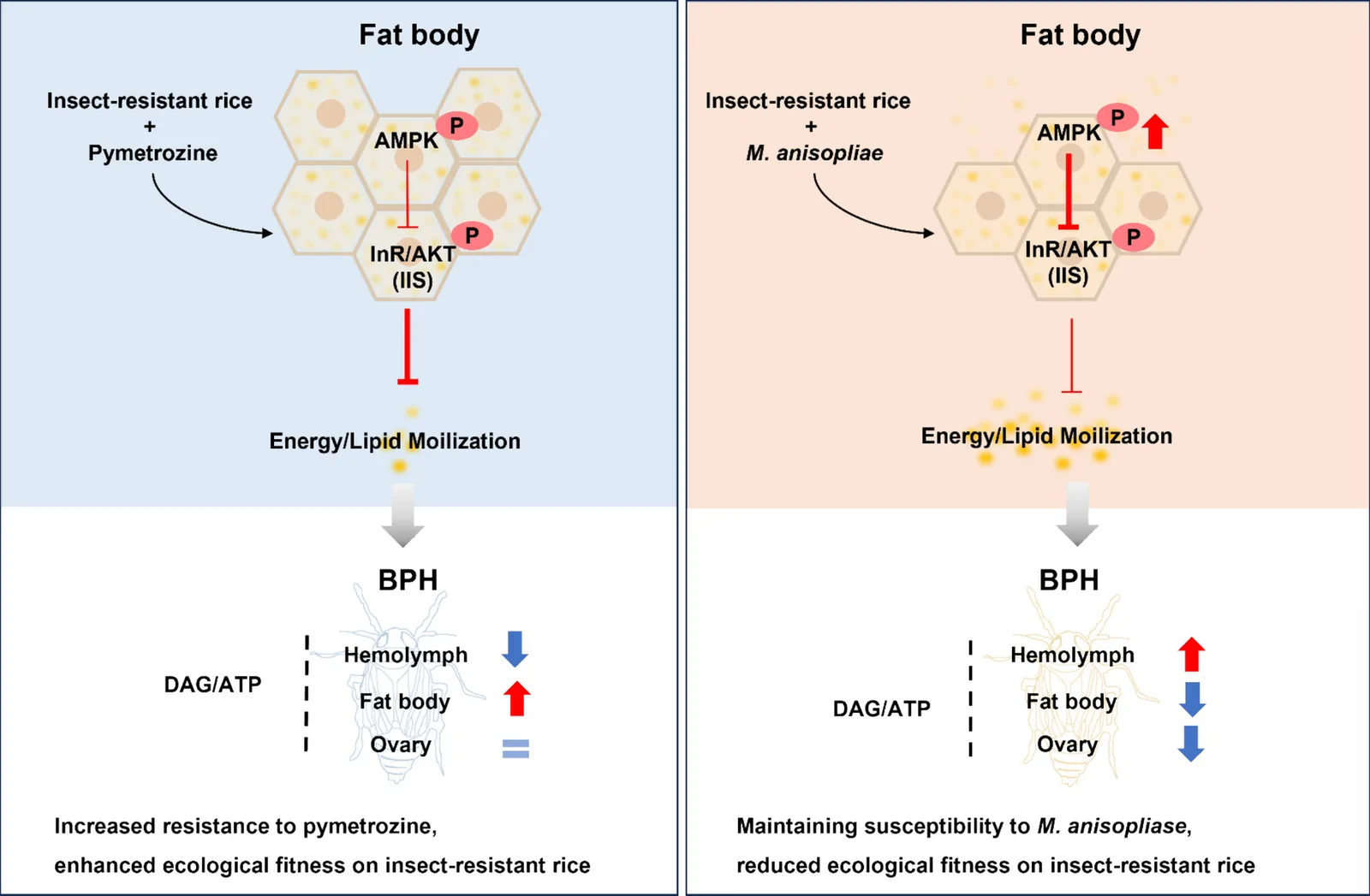
Mechanistic model illustrating how the integrated application of insect-resistant rice IR36 and *M. anisopliae* CQMa421 achieves sustained control of the BPH. Combined exposure to insect-resistant rice IR36 and *M. anisopliae* CQMa421 activates AMPK phosphorylation in the fat body of BPHs, which in turn suppresses the insulin signaling pathway (IIS) by reducing the phosphorylation of InR and AKT and inhibits lipid degradation, with mobilized energy substrates preferentially redistributed to the hemolymph to support humoral immunity. As a result, energy reserves in the fat body are reduced, reducing P450-mediated detoxification capacity and overall ecological fitness in resistant rice. The limited energy in the hemolymph is insufficient to counteract fungal infection, maintaining BPH susceptibility to *M. anisopliae* CQMa421. Together, this energy allocation strategy underlies the long-term efficacy of the combined plant resistance and microbial control approach, providing a mechanistic basis for sustainable pest management strategies

In conclusion, this study demonstrates that the combination of insect-resistant rice and *M. anisopliae* CQMa421 achieves sustainable control of BPHs under laboratory conditions through a coordinated mechanism involving AMPK activation, IIS suppression, and systemic lipid redistribution. Energy mobilization prioritizes humoral immunity, reducing pest fitness and maintaining susceptibility to both resistant rice and microbial pathogens (Fig. 7). Our findings contribute mechanistic insights into energy-mediated pest adaptation and provide a potential foundation for extending the longevity and efficacy of resistant crop varieties. Future research should focus on field validation, multi-pest applicability, and exploration of additional metabolic and immune pathways that may enhance sustainable pest management strategies.

## Materials and methods

### Insects

The BPH population used in this study was originally collected from a rice field in Guangzhou, Guangdong Province, China. The population was subsequently maintained on the susceptible rice variety Taichung Native 1 (TN1) for more than five years under controlled laboratory conditions [26 ± 2 °C, 80 ± 10% relative humidity (RH), and a 16:8 h light–dark photoperiod] without exposure to insecticides or resistant rice varieties. This colony was designated the P-TN1 population.

To develop a population adapted to the insect-resistant rice variety IR36, individuals from the P-TN1 population were subjected to continuous selection on IR36 for 60 generations. This process resulted in a highly adapted population, referred to as the P-AD population. The ecological fitness of the P-AD population on the resistant rice variety IR36 did not differ significantly from that of the P-TN1 population on the susceptible TN1 variety. In parallel, a subset of the P-TN1 population was continuously maintained on TN1 and used as the control population throughout the experiment. Colonies were maintained at 26 ± 2 °C, 80 ± 10% RH, 16:8 h L:D; bioassays were conducted at 27 ± 1 °C, 80 ± 5% RH, 16:8 h L:D.

### Insecticides

Technical-grade active ingredients of imidacloprid (96%), dinotefuran (97%), fluazinam (97%), thiamethoxam (98%), acetamiprid (96%), and clothianidin (99%) were obtained from Syngenta AG (Basel, Switzerland). The entomopathogenic fungus *M. anisopliae* CQMa421, formulated as a dispersible oil suspension, was purchased from Chongqing Julixin Bioengineering (Chongqing, China).

The active ingredient chlorpyrifos (98%) was obtained from Foshan Yinghui Crop Science (Foshan, China). Fluazinam (92%) was additionally sourced from Shenzhen Noposion Agrochemicals (Shenzhen, China). The active ingredient pymetrozine (95%) was purchased from Sinochem Ningbo (Ningbo, China).

### Evaluation of insecticide resistance in the P-AD population

To evaluate insecticide resistance in the P-AD population of BPH, bioassays were conducted using multiple insecticides at different concentrations. The insecticides imidacloprid, dinotefuran, fluazinam, acetamiprid, clothianidin, nitenpyram, chlorpyrifos, and pymetrozine were tested at concentrations of 12.5, 25, 50, 100, and 200 mg/L. Thiamethoxam was tested at concentrations of 2, 4, 8, 16, and 32 mg/L. For the entomopathogenic fungus *M. anisopliae* CQMa421, spore suspensions were prepared at concentrations of 8 × 10^5^, 4 × 10^6^, 2 × 10^7^, 1 × 10^8^, and 5 × 10^8^ conidia/mL.

The susceptibility of BPH to these insecticides was assessed using a rice seedling immersion method. Each insecticide was first dissolved in an appropriate volume of acetone and then diluted with deionized water to obtain the desired concentrations. Subsequently, 1% Tween-80 was added as a surfactant.

Rice seedlings (TN1) were immersed in the respective insecticide solutions for 30 s, while deionized water served as the control treatment. After treatment, the seedlings were allowed to air-dry and were then placed individually into glass tubes. Thirty third-instar BPH nymphs were introduced into each tube, and the experiment began once all nymphs had climbed onto the rice plants. If mechanical losses resulted in fewer than thirty individuals per replicate, the missing individuals were replaced immediately.

All bioassays were conducted under controlled environmental conditions (27 ± 1 °C, 80 ± 5% RH, and a 16:8 h light–dark photoperiod). Mortality was recorded 96 h after treatment with chemical insecticides and 144 h after treatment with *M. anisopliae* CQMa421.

Dose–response regression equations were established to evaluate the toxicity of the tested insecticides to BPH populations. The median lethal concentration (LC_50_) values and their corresponding 95% confidence intervals were calculated. Based on these values, resistance ratios (RRs) were determined for the P-AD population relative to the control population.

### Changes in insecticide resistance and adaptation to resistant hosts in the P-TN1 population following multiple generations of exposure to sublethal insecticides

To investigate how continuous multi-generational exposure to sublethal concentrations of different insecticides affects insecticide resistance and host adaptation in BPH, experiments were initiated using the P-TN1 population (Fig. 1A). Briefly, two hundred pairs of one-day-old female and male adults were selected for mass mating to generate the G0 generation. The resulting G0 nymphs were reared on the susceptible rice variety TN1 until they reached the third-instar stage.

At this stage, nymphs were treated with one of three insecticides: *M. anisopliae* CQMa421 (1.75 × 10^6^ conidia/mL), pymetrozine (17.48 mg/L), or thiamethoxam (1.98 mg/L). Each compound was applied at the LC_10_ concentration previously determined for the P-TN1 population using a direct spraying method. After three days of exposure, surviving insects were transferred to untreated rice plants and reared until adulthood.

Newly emerged one-day-old female adults were subsequently evaluated for insecticide resistance levels and for their biological performance on the resistant rice variety IR36. Specifically, survival rate, honeydew excretion, and total offspring production were assessed.

### Biological assays to measure survival, honeydew excretion, and total offspring production

Survival was evaluated by placing thirty one-day-old female adults on healthy, tillering-stage plants of the insect-resistant rice variety IR36. After three days of feeding, the number of surviving individuals was recorded. Each treatment group contained thirty insects, and all assays were conducted with three biological replicates.

Honeydew production was measured to assess feeding activity. One-day-old female insects were individually placed in small parafilm bags (5 cm × 3 cm) that were weighed prior to the experiment. Each bag containing a BPH individual was attached to a rice stem at approximately 2 cm above the soil surface and allowed to feed for 72 h. At the end of the feeding period, the bags were removed and weighed again. Honeydew production was calculated as the difference between the bag weight before and after feeding. Each bag represented one biological replicate, and at least 20 replicates were included per treatment.

To assess reproductive performance, unmated adults were paired at a 1:1 male-to-female ratio. Each treatment group consisted of ten replicates. After successful mating and the subsequent hatching of progeny, the number of offspring was recorded. In addition, oviposited rice plants were dissected to count unhatched eggs. This allowed the calculation of the total number of offspring produced per female.

### Evaluation of the multi-generational control effect of combining insect-resistant rice with different types of pesticides

To evaluate the lethal effect of IR36 in combination with different insecticides, *M. anisopliae* CQMa421 and pymetrozine were applied at their respective LC_50_ concentrations (Fig. 2A).

A total of 2000 pairs of one-day-old P-TN1 adults were first allowed to mate to generate sufficient G_0_ first-instar nymphs. These nymphs were continuously maintained on the insect-resistant rice variety IR36. When the nymphs reached the third-instar stage, they were treated with either *M. anisopliae* CQMa421 or pymetrozine at their respective LC_50_ concentrations. Mortality was recorded seven days after treatment.

Surviving insects from both treatment groups were collected after emergence as one-day-old female adults. These adults were then evaluated for insecticide resistance levels, honeydew excretion, and total offspring production on IR36 rice plants.

From the surviving population, 200 pairs of female and male adults were selected for mass mating to generate the next generation. This procedure was repeated across successive generations to assess the long-term dynamics of pest adaptation and the sustained control efficacy of the combined management strategy.

### Metabolite determination

The concentrations of triglycerides (TAG), diacylglycerol (DAG), and adenosine triphosphate (ATP) were determined using commercial ELISA kits (Jiangsu Yutong, China) following the manufacturer's instructions. Briefly, an appropriate amount of sample was collected and homogenized in phosphate-buffered saline (PBS), after which the homogenate was centrifuged to obtain the supernatant. Subsequently, the protein concentration of the collected supernatant was determined using the BCA assay, and the samples were finally adjusted to a protein concentration of 1 mg/mL. The supernatant was then used for the colorimetric reaction according to the kit protocol. Absorbance was measured at 450 nm using a ROCHE-P450 automatic biochemical analyzer (Roche, Basel, Switzerland). Concentrations of metabolites were calculated based on the obtained optical density (OD) values following the method described by Lin et al. [67].

### Eosin Y staining

Neutral lipid distribution in the epidermis was examined using Eosin Y staining following the method described by Wang et al. [75]. Briefly, BPH specimens were incubated in a staining solution containing 0.5% Eosin Y (w/v) and 0.1% Triton X-100. The staining was conducted at room temperature for 30 min. After incubation, the specimens were washed three times with distilled water to remove excess dye and subsequently observed under a microscope.

### Lipid droplet staining

Fat body tissues were dissected from BPH individuals and fixed in 4% paraformaldehyde at room temperature for 30 min in the dark. After fixation, the samples were stained with BODIPY to visualize lipid droplets to label nuclei. The staining reaction was carried out at room temperature for 30 min. Subsequently, the samples were washed three times with PBS, with each wash lasting 5 min. Finally, the tissues were mounted using mounting medium and observed under a fluorescence microscope following the protocol described by Lin et al. [67].

### RNA-seq

One-day-old female BPHs from two treatment populations (P-IR36_Pym and P-IR36_Ma) were collected after 13 generations of exposure to different insecticides in combination with the resistant rice variety IR36. Each treatment group consisted of three biological replicates, with thirty insects per replicate.

Total RNA was extracted using the MicroElute® Total RNA Kit according to the manufacturer’s instructions. The extracted RNA samples were sent to Guangzhou Biomarker Technologies for library construction and sequencing. Library quality was assessed prior to sequencing using the Illumina NovaSeq 6000 platform.

After passing quality control, paired-end sequencing was performed using the PE150 mode. The raw sequencing data were subsequently processed to generate high-quality clean reads. These reads were aligned to the reference genome (ASM1435652v1) to obtain mapped data. Downstream analyses included sequence quality control, library quality evaluation, transcript structural analysis, differential gene expression analysis, gene expression profiling, functional enrichment analysis of differentially expressed genes, and KEGG pathway annotation. Differentially expressed genes (DEGs) were identified using DESeq2, with thresholds of |log2FC| ≥ 1 and FDR-corrected *P* < 0.05.

### Quantitative real-time PCR (RT-qPCR) analysis

One-day-old female insects from the two populations (P-IR36_Pym and P-IR36_Ma), as well as their respective tissues, were collected for RT-qPCR analysis. RNA extraction, reverse transcription, and quantitative PCR procedures were performed following the protocols described by Lin et al. [67].

Relative gene expression levels were calculated using the 2^−△△Ct^ method. The housekeeping gene β-actin was used as the internal reference to normalize gene expression levels. The sequences of primers used in the analysis are listed in Table S1.

### Western blot

Total protein was extracted using NP40 lysis buffer, and the protein concentration was determined using the bicinchoninic acid (BCA) method following the protocol described by Chen et al. [76]. The protein samples were subsequently diluted to a concentration of 1 μg/μL. Proteins were separated by SDS-PAGE using a 10% resolving gel and a 5% stacking gel. A volume of 10 μL of protein sample (corresponding to 10 μg total protein) was loaded into each well.

After electrophoresis, proteins were transferred onto membranes and subjected to immunoblotting using the following primary antibodies: anti-AMPKα (Abcam, 1:1000), anti-phospho-AMPKα (CST, 1:2000), anti-AKT (CST, 1:2000), anti-phospho-AKT (CST, 1:2000), anti-phospho-InR (Abcam, 1:4000), anti-Vg (CST, 1:5000), and anti-β-actin (Abcam, 1:10000).

HRP-conjugated goat anti-rabbit IgG (TransGen Biotech, 1:10000) was used as the secondary antibody. Protein bands were visualized using enhanced chemiluminescence (ECL) reagents (Yeasen Biotech, Shanghai, China) and detected using a chemiluminescence imaging system.

### Enzyme activity measurement

Enzyme activity was determined using a commercial detection kit (Jiangsu Yutong, China) according to the manufacturer’s instructions based on an ELISA method. Briefly, samples were homogenized and incubated with NP40 lysis buffer for 60 min at 4 °C. The homogenates were then centrifuged, and the resulting supernatants were collected for ELISA.

Absorbance values (OD) were measured at 480 nm using a ROCHE-P450 automatic biochemical analyzer (Roche, Basel, Switzerland). Each treatment included at least five independent biological replicates [77].

### RNA interference (RNAi)

Double-stranded RNA (dsRNA) was synthesized using the MEGAscript T7 High Yield Transcription Kit (Promega, Madison, WI, USA) following the method described by Chen et al. [78]. In this study, dsAMPKα and dsGFP were generated. The quality and integrity of the synthesized dsRNA were verified by agarose gel electrophoresis, and the concentration was measured using a NanoDrop 2000 spectrophotometer (Thermo Fisher Scientific, Waltham, MA, USA).

Using dsGFP as a control, 200 ng of dsRNA was injected into the BPH. Surviving individuals were used for subsequent experiments. The sequences of primers used for dsRNA synthesis are listed in Table S1.

### Chemical compound injection experiment

The compounds 5-aminoimidazole − 4-carboxamide − 1-β-D-ribonucleoside (AICAR; Selleck, Shanghai, China) and Compound C (CC; InvivoChem, USA) were dissolved in dimethyl sulfoxide (DMSO; Selleck, China) to prepare working solutions at the desired concentrations.

Following the injection procedure used in the RNAi experiments, the prepared solutions were injected into the hemocoel of one-day-old female adults. Surviving BPHs were subsequently used for further experiments.

### Rescue assay

To verify the specificity of the *NlAMPKα* knockdown phenotype, a rescue assay was performed. Briefly, 200 ng of dsRNA was injected into BPH, and the surviving individuals were used for subsequent experiments. Two days after RNAi treatment, the surviving insects were injected with 200 ng of AICAR. Two days after AICAR injection, the surviving BPHs were used for further experiments, and the relevant phenotypes and biochemical parameters were recorded and compared among the different treatment groups. Each treatment was performed with at least three independent biological replicates.

### Statistical analysis

Estimation of LC_50_ and resistance ratios. Median lethal concentrations (LC_50_) and their 95% confidence limits (CLs) were estimated using SPSS version 27.0 (IBM, Chicago, IL, USA). The resistance ratio (RR) was calculated as LC_50_ (adapted population, P-AD)/LC_50_ (control population, P-TN1). LCRs and their corresponding 95% CLs were calculated to compare toxicity between treatments; differences between LCRs were considered statistically significant when the 95% CL did not include 1 [79].

Linear regression across generations. Simple linear regression was used to test whether biological performance traits changed monotonically across successive generations of continuous exposure, with generation number as a continuous predictor and each trait as the response variable. Separate models were fitted for each population (P-Ma, P-Pym, P-Thi, P-IR36_Ma and P-IR36_Pym). Slope significance was assessed using a two-tailed t-test (equivalent to the F-test) at *α* = 0.05, and goodness of fit was quantified by *R*^2^ and adjusted *R*^2^. Residual diagnostics confirmed linearity, normality (Shapiro–Wilk test, *P* > 0.05) and homoscedasticity (Breusch-Pagan test, *P* > 0.05). Linear fitting was appropriate because no prior nonlinear model was hypothesized, the generation range was limited, residual assumptions were satisfied, and the slope directly estimated the per-generation rate of change. *P* < 0.05 was considered statistically significant.

Pharmacological, RNAi and rescue assays. In the pharmacological, RNAi and rescue assays, all data are presented as means ± SEM unless otherwise stated. Statistical analyses were performed using one-way analysis of variance (ANOVA) followed by Tukey's honestly significant difference (HSD) post hoc test for multiple comparisons among the six treatment groups (DMSO, CC, AICAR, dsGFP, ds*NlAMPKα* and ds*NlAMPKα* + AICAR). The significance level was set at *α* = 0.05. Different letters indicate statistically significant differences between groups.

Western blot bands were quantified using ImageJ software. Relative protein expression was calculated as the ratio of the target band to the loading control and normalized to the control group. Saturated bands were excluded, and all measurements were performed under identical conditions.

## Supplementary Information

Supplementary Material 1: Fig. S1 Knockdown efficiency of *NlAMPKα* in the fat body of BPH populations P-IR36_Pym and P-IR36_Ma. Fig. S2 Expression levels of *NlAMPKα*, *NlAMPKβ* and *NlAMPKγ* in P-IR36_Pym and P-IR36_Ma populations of BPHs. Table S1 The primers used in this study. Table S2 Cross-resistance of the adapted population (P-AD) to other insecticides, which are frequently used on *Nilaparvata lugens*. Table S3 Differential genes in the transcritomes of BPH populations P-IR36_Pym and P-IR36_Ma.

## Data Availability

The raw RNA-seq data generated in this study have been deposited in the NCBI Sequence Read Archive (SRA) under BioProject accession number PRJNA1449468.
