# Supplementary material for: Insect-resistant rice and Metarhizium anisopliae sustain the brown planthopper susceptibility through AMPK-mediated lipid redistribution
Source: Crop Health. 2026 Nov 14;4(1):28. doi: 10.1007/s44297-026-00090-7 (PMC13769798; doi:10.1007/s44297-026-00090-7)
Supplement: Supplementary file 1 [file 44297_2026_90_MOESM1_ESM.zip › Supplementary Information.docx]

**Supplementary materials**

**Figure S1. Knockdown efficiency of *NlAMPKα* in the fat body of BPH populations P-IR36_Pym and P-IR36_Ma.** **Relative mRNA expression of *NlAMPKα* in the fat body of adult female BPHs at 48 h after injection with ds*NlAMPKα* or dsGFP (control), as determined by RT-qPCR..**. Data are presented as means ± SEM (n = 3 biological replicates). Double asterisks ** indicate extremely significant (*P* < 0.01) differences, based on pairwise comparisons using Student’s t-test.

**Figure S2. Expression levels of *NlAMPKα*, *NlAMPKβ* and *NlAMPKγ* in P-IR36_Pym and P-IR36_Ma populations of BPHs.**

**Table S1.** The primers used in this study.

| **Primers** | **Sequences (5’ to 3’)** |
| --- | --- |
| *NlAMPKα*-qF | GGTGACATCGCTGGTGAATC |
| *NlAMPKα*-qR | GGGGCAAGGTCGATCACTA |
| *NlAMPKβ*-qF | CGACGATGAGGAACCGTATT |
| *NlAMPKβ*-qR | GGATGGTAGAGTTAGTGTAAAACGACA |
| *NlAMPKγ*-qF | CGGTTCTATCGTGCAATGTC |
| *NlAMPKγ*-qR | TGCAATACTAGATTAGCGAATGGTT |
| *Actin-qF* | TGCGTGACATCAAGGAGAAGC |
| *Actin-qR* | CCATACCCAAGAAGGAAGGCT |
| *NlAMPKα-*dsRNA-F | AGACTTTGGGAGTAGGAACT |
| *NlAMPKα-*dsRNA-R | CTTGTTCTCAGAAACTCTCCA |
| T7-*NlAMPKα*-dsRNA -F | GGATCCTAATACGACTCACTATAGGAGACTTTGGGAGTAGGAACT |
| T7-*NlAMPKα*-dsRNA -R | GGATCCTAATACGACTCACTATAGGCTTGTTCTCAGAAACTCTCCA |
| GFP-*-*dsRNA-F | AAGGGCGAGGAGCTGTTCACCG |
| GFP-*-*dsRNA-R | CCATACGGAATCCATCCACAC |
| T7-dsGFP-dsRNA -F | GGATCCTACTATAGGAAGGGCGATACGACTCAAGGAGCTGTTCACCG |
| T7-dsGFP-dsRNA -R | GGATCCTAATACGAGGCAGCAGGACCATGTGAACTCACTATTCGCGC |

**Table S2.** Cross-resistance of the adapted population (P-AD) to other insecticides, which are frequently used on *Nilaparvata lugens*.

| **Insecticide** | **Population** | **LC_50_ (95% CI)**  **(mg/mL)** | **Slope (SE)** | **χ^2^(df)** | **RR ^a^** | **LCR ^a^** |
| --- | --- | --- | --- | --- | --- | --- |
| Acetamiprid | P-TN1 | 40 (31-50) | 2.48 | 2.08 (3) | - | - |
|  | P-AD | 77 (58-107) | 1.87 | 1.77 (3) | 1.92 | 0.52 (0.42-0.65) * |
| Dinotefuran | P-TN1 | 37 (29-46) | 2.62 | 2.84 (3) | - | - |
|  | P-AD | 86 (64-126) | 1.78 | 0.54 (3) | 2.33 | 0.43 (0.34-0.54) * |
| Clothianidin | P-TN1 | 43 (34-53) | 2.54 | 1.71 (3) | - | - |
|  | P-AD | 117 (83-198) | 1.59 | 0.24 (3) | 2.74 | 0.49 (0.40-0.62) * |
| Nitenpyram | P-TN1 | 40 (31-50) | 2.38 | 2.58 (3) | - | - |
|  | P-AD | 54 (42-69) | 2.30 | 0.64 (3) | 1.362 | 0.73 (0.61-0.89) * |
| Imidacloprid | P-TN1 | 39 (30-49) | 2.35 | 0.79 (3) | - | - |
|  | P-AD | 135 (90-269) | 1.40 | 0.31 (3) | 3.46 | 0.29 (0.21-0.40) * |
| Chlorpyrifos | P-TN1 | 55 (23-160) | 2.19 | 7.87 (3) | - | - |
|  | P-AD | 51 (23-118) | 2.20 | 6.68 (3) | 0.928 | 1.07 (0.87-1.31) |
| Fluazinam | P-TN1 | 65 (51-85) | 2.17 | 0.88 (3) | - | - |
|  | P-AD | 115 (85-180) | 1.82 | 1.15 (3) | 1.78 | 0.56 (0.44-0.72) * |

LCR (lethal concentration ratio) is the ratio of the LC50 of the adapted population (P-AD) to that of the control population (P-TN1); if this 95% confidence interval does not contain the value 1, the result is considered statistically significant at the 5% level and is marked with an asterisk (*).
